# Impact of maintenance anaesthetics on total emissions of an atrial fibrillation catheter ablation procedure—Authors’ reply

**DOI:** 10.1093/europace/euad355

**Published:** 2023-11-28

**Authors:** Geoffroy Ditac, Rémi Schweizer, Pierre-Jean Cottinet, Francis Bessière

**Affiliations:** Department ofElectrophysiology, Hôpital Cardiologique Louis Pradel, Hospices Civils de Lyon, 28 avenue du Doyen Lépine, 69500 Bron, France; Department of Anesthesia and ICU, Hôpital Cardiologique Louis Pradel, Hospices Civils de Lyon, Lyon, France; LGEF, INSA-Lyon, EA682, University Lyon, 69621 Villeurbanne, France; Department ofElectrophysiology, Hôpital Cardiologique Louis Pradel, Hospices Civils de Lyon, 28 avenue du Doyen Lépine, 69500 Bron, France

We thank Dr Kalmar *et al.*^1^ for their insightful comments on our study on the carbon footprint of atrial fibrillation (AF) catheter ablation.^2^ We really appreciate the comparison of the global warming potential (GWP) of different general anaesthesia practices.

As rightly pointed out, our study primarily addressed the operator-related aspects of the procedure. However, the importance of the anaesthetic component should not be underestimated, with an average emission of 19.7 kg of CO_2_ equivalent (CO_2_-e) per procedure, constituting 25.6% of the total emissions. The primary contributor (54.5%) to this footprint was the use of anaesthetic gas, drugs, and their administration. This aspect will deserve further precision in the discussion.

In our hospital, all electrophysiology procedures requiring transseptal access are performed under general anaesthesia due to considerations of efficacy and safety. Although alternatives such as deep or conscious sedation are available, the optimal anaesthesia strategy for achieving the best clinical outcomes remains unclear.^3–5^ Furthermore, general anaesthesia currently stands as the most widely used approach,^6^ a trend likely to persist with the development of pulsed-field ablation.

Historically, our anaesthesiologists have favoured halogenated gas for general anaesthesia because of its presumed cardioprotective properties in cardiac surgery.^7^ As highlighted by Kalmar *et al.* and supported by previous studies,^8^ desflurane results in massive greenhouse gas (GHG) emissions, exhibiting a 100-year GWP ∼20 times higher than sevoflurane. Recognizing this significant environmental impact, our institution ceased to use desflurane in 2021, opting exclusively for sevoflurane.

Consequently, all patients reported in our study underwent general anaesthesia with sevoflurane, consuming an average of 19.4 mL per procedure. While acknowledging the relevance of a 20-year horizon for anaesthetic gases, we retained the 100-year horizon in line with the usual methodology of European and USA environmental agencies. Using the GWP-100 methodology, the mean GHG emissions for sevoflurane were calculated as 3.8 kg of CO_2_-e (excluding packaging and transportation). Discrepancies with the estimates of Kalmar *et al.* emphasize the complexity of GWP calculations, reinforcing the necessity for standardized methodologies and databases for eco-audits in the healthcare sector.^8^

The remaining carbon footprint of the anaesthetic component was mainly due to injectable drugs (propofol, midazolam, ketamine, etc.). For each injectable product used, we sought direct information on the synthesis mechanisms. In the absence of available data, we modelled each step of the reaction using the ‘SciFinder’ chemistry research platform. Again, the lack of a reference database makes this approach less robust and may explain differences in conclusions and interpretations when compared to other studies. In the end, the exact number is not as important as the order of magnitude, which enables priority actions to be identified.

Importantly, CO_2_-e is not the sole concern. Although more complex, especially for pharmaceuticals, a comprehensive life cycle analysis should be systematically carried out. For instance, intravenous propofol is associated with low GHG emissions but is responsible for water and soil contamination.^9^

Finally, we underscore the importance of optimizing general anaesthesia settings to improve sustainability. In an analysis of over a thousand AF ablations performed in our centre during the last 5 years, a significant trend towards reduced gas consumption has been observed. However, there is still variability between procedures (median 25 mL, interquartile range 17–35 mL), depending on operator settings. The French Society of Anesthesia and Intensive Care (SFAR) recently published guidelines on reducing the environmental impact of general anaesthesia,^10^ recommending the use of a low fresh gas flow, automated control of end-tidal gas concentrations, and systematic monitoring of anaesthesia depth to adjust gas or drug concentrations. While gas recapture is mentioned, no specific recommendation is provided due to insufficient data.

In conclusion, promoting changes in anaesthesia practice is clearly an important way to improve the sustainability of electrophysiology procedures. This transformation should follow the principles of ‘reduce, reuse, recycle’, without compromising the effectiveness and safety of patient care.
